# Ultra-Broadband and Compact TM-Pass Polarizer Based on Graphene-Buried Polymer Waveguide

**DOI:** 10.3390/polym14071481

**Published:** 2022-04-06

**Authors:** Baizhu Lin, Tianhang Lian, Shijie Sun, Mu Zhu, Yuanhua Che, Xueqing Sun, Xibin Wang, Daming Zhang

**Affiliations:** State Key Laboratory of Integrated Optoelectronics, College of Electronic Science and Engineering, Jilin University, Changchun 130012, China; linbz19@mails.jlu.edu.cn (B.L.); lianth19@mails.jlu.edu.cn (T.L.); sunsj20@mails.jlu.edu.cn (S.S.); zhumu20@mails.jlu.edu.cn (M.Z.); cheyh21@mails.jlu.edu.cn (Y.C.); xqsun21@mails.jlu.edu.cn (X.S.); zhangdm@jlu.edu.cn (D.Z.)

**Keywords:** integrated optics, polymer optical waveguides, optical communication systems, monolayer graphene, polarizer

## Abstract

We report an ultra-broadband and compact TM-pass polarizer based on graphene-buried polymer waveguides. The characteristic parameters of the polarizer were carefully designed and optimized. The standard microfabrication processes were employed to fabricate the device. The presented polarizers exhibit high polarization-dependent transmission imposing a TE mode cutoff while leaving the TM mode almost unaffected. We experimentally demonstrated the polarizer that has an ultra-high extinction ratio of more than 22.9 dB and 41.9 dB for the monolayer graphene film placed on the surface of core layer and buried in the center of core layer, respectively, and as low insertion loss as ~4.0 dB for the TM mode with the bandwidth over 110 nm. The presented polarizer has the advantages of high extinction ratio, ultra-broadband, low cost, and easy integration with other polymer-based planar lightwave devices.

## 1. Introduction

Polarization state control of the light has important applications in optical communications and photonic integrated circuits, especially for the systems with only one operating polarization [1]. Optical polarizer is one of the key elements in the optical communication system and plays an important role, because many optical components have requirements for the polarization states, e.g., the transverse electric (TE) or the transverse magnetic (TM) polarization modes [2,3,4,5,6]. For example, the electro-optical modulators based on polymer materials often work under TM-polarization [7,8]. The functionality of the optical polarizer is to allow one polarization to pass (TE or TM) and the other to be eliminated. In recent years, researchers have implemented many types of optical polarizers, including prism polarizer, sheet polarizer, optical fiber polarizer and optical waveguide polarizer [3,4,9,10]. Compared with other types of polarizers, optical waveguide polarizer is flexible and easy to integrate with other optical components on one circuit. A number of optical polarizers based on different waveguide structures have been developed, including hybrid plasmonic waveguides [11,12], photonic crystal waveguides [13], silicon slot waveguides [14,15,16] and silicon/graphene hybrid waveguides [17,18]. However, they are mainly designed for silicon photonics applications, and many of them are still at the stage of theoretical design and simulation. In recent years, polymer photonics have attracted broad attention because of their excellent properties such as easy integration, low cost, and multi-function realized with the material engineering, which make polymer materials an ideal platform for developing the complex optical devices and systems [19,20,21,22]. Especially, to fabricate photonic integrated devices and circuits with low intrinsic losses, mechanical flexibility, and large surfaces, polymer waveguides can offer a very attractive solution with the potential of using simple, fast, and cost-competitive manufacturing procedures [23,24]. With the development of polymer photonics, it is essential to carry out polymer waveguide-based polarizers with low cost and simple fabrication process, and more importantly have low insertion loss, high extinction ratio and large bandwidth.

Here, we report an ultra-broadband and compact TM-pass polarizer based on graphene-buried polymer waveguides. The polarization mechanism is mainly due to the differential attenuation of the two polarization modes in the presented hybrid waveguides. The change of the modal characteristics for the TE mode is more than that of the TM mode, which is derived from the fact that the electric field of the TE mode interacts with graphene more effectively than that of the TM mode. Moreover, thanks to the flexible fabrication process of the polymer waveguide, the graphene film can be easily placed anywhere inside the waveguide by using the standard microfabrication process. The presented polarizers have the advantages of low insertion loss, high extinction ratio, and easy integration with other polymer-based photonic integrated devices.

## 2. Device Design and Simulation

**Figure 1:**
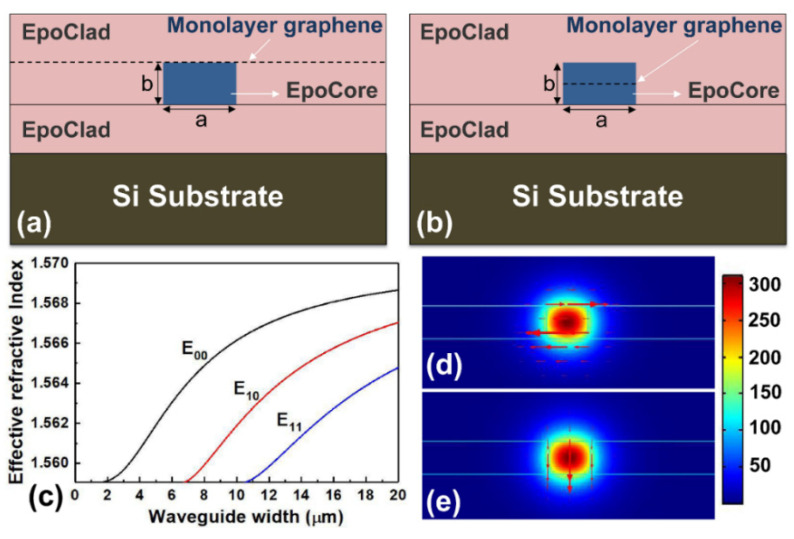
Schematic diagram of the polarizer with the monolayer graphene film placed (**a**) on the surface of core layer and (**b**) buried in the center of core layer. (**c**) Relations between effective indices *N_eff_* of the waveguide and core width. The simulated intensity patterns of the fundamental mode with the optimized core dimension for (**d**) TE and (**e**) TM polarizations, respectively.

Polarizers with the graphene layer buried in different locations are designed to examine the effects of the buried location on their loss performance. The schematic structures of the demonstrated two types of polarizers are shown in Figure 1. In Figure 1a,b, the monolayer graphene film was placed on the surface of core layer and in the center of core layer, respectively. Before analyzing the loss performance of the device, the dimension of the waveguide, defined structure, and refractive index of waveguide material were tailored carefully to achieve single-mode transmission in the waveguide. Therefore, in the following calculation and optimization, the refractive index of waveguide core and cladding are selected to be 1.570 and 1.559, respectively, the free-space wavelength *λ*_0_ is 1550 nm. The relations between the waveguide core width *a* and mode effective indices *N_eff_* of the strip waveguide without a graphene layer is shown in Figure 1c, where the waveguide thickness *b* = 0.8*a*. In order to realize single-mode transmission in the waveguide, the core width *a* should be selected in the range of 2~7 µm, meanwhile, the core thickness *b* was chosen in the range of 1.6~5.6 µm. The simulated intensity patterns of the fundamental mode with the optimized core dimension for TE and TM polarizations are shown in Figure 1d,e, respectively.

**Figure 2:**
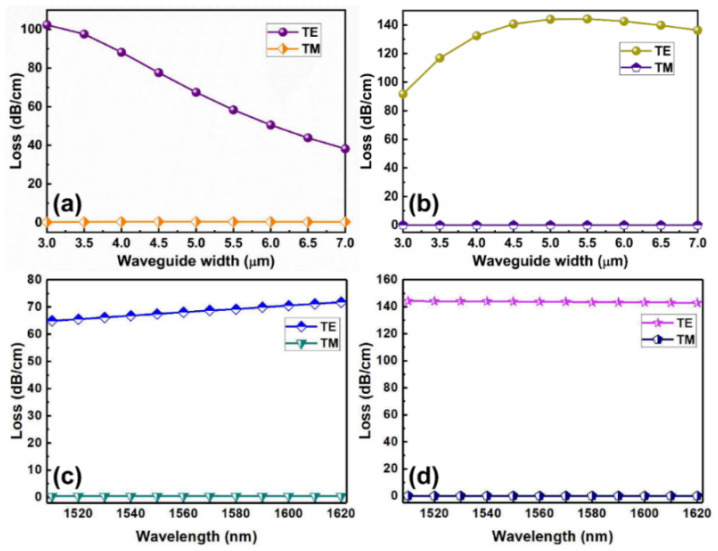
Variation of the change in the modal loss induced by the graphene film with the core dimension when the graphene film was placed (**a**) on the surface of core layer and (**b**) buried in the center of core layer. The fundamental mode loss characteristics of graphene films (**c**) on the surface of core layer and (**d**) buried in the center of core layer in the hybrid waveguides.

The complex effective indices of the hybrid polymer waveguide embedded with graphene layer were calculated based on the interface model by using the finite-element method with the commercial full-vector mode solver COMSOL. In the simulation, we have established triangle meshes with a maximum mesh length of 500 nm and applied perfect matched layers as the boundary condition, which can help us get an accurate numerical result since electromagnetic waves are perfectly absorbed in the boundary without reflections. The modal loss (i.e., mode power attenuation, *MPA*) can be calculated with the imaginary part of the effective refractive index according to the following formula [25]:

(1) MPA=40πlog(e)Im(Neff)/λ0

where Im(*N_eff_*) is the imaginary part of the effective mode index of the waveguide. The modal loss induced by the graphene was affected by the core dimension, which is mainly due to the effect of the core dimension on the modal field intensity distributions in the waveguide. As shown in Figure 2a, with the increase of the core dimension, the modal loss induced by the graphene film buried on the surface of the core gradually decreases for the TE polarization, while the TM mode maintains a lower loss. It is mainly because the modal field intensity distributions at the interface between core and cladding will be decreased with the increase of the core dimension. Figure 2b shows the modal loss induced by the monolayer graphene buried in the center of core with the core dimension. As shown in Figure 2b, for the TE polarization, the modal loss increase with an increase in the core dimension up to a maximum value and then decreases with a further increase in core dimension. The maximum change in the modal loss occurs at a waveguide width of 5.0 µm. This can be explained that the modal field intensity distribution in center of core increase with the increase of the core dimension, therefore, there is a stronger interaction between TE mode and graphene layer. However, as the core dimension further increases, the modal field intensity distribution inside the core layer begins to diverge, and the modal field intensity in the in center of core begins to weaken, therefore, the interaction between TE mode and graphene layer weakens. Considering the coupling efficiency between the device and optical fiber and the performance of the devices, the core width and thickness were finally selected with 5.0 µm and 4.0 µm, respectively. Figure 1d,e show the simulated intensity patterns of the fundamental mode for TE and TM polarizations, respectively, with the optimized core dimension.

Figure 2c,d show the loss performance of fundamental mode induced by the graphene film in the hybrid waveguides characterized in the wavelength range of 1510~1620 nm, which are corresponding to the structures shown in Figure 1a,b. It can be seen that the graphene-induced modal losses are highly polarization dependent: the TM mode has an ultra-low loss, while the TE mode presents a larger loss, and the graphene-induced loss is insensitive to the wavelength. By contrasting the loss between TM and TE modes, an efficient TM-pass polarizer can be built with both high extinction ratio and low insertion loss. It is also clear that the locations of the buried graphene film have an important effect on the loss performance of the hybrid waveguide. The modal loss induced by the graphene embedded in the center of core is obviously larger than that induced by the graphene buried on the surface of core. The fact that the graphene film has a much stronger influence on the TE mode than the TM mode of the hybrid waveguide can be easily understood by the modal field intensity distributions. For the TE modes, the major electric-field components are confined in the center of the core layer and tangential to the buried graphene film surface, then, have a stronger graphene–light interaction. Therefore, the conductivity of the graphene can significantly attenuate the electric field. However, for the TM mode, the major electric-field components confined in the center of core are normal to the graphene film surface, and the conductivity of the graphene cannot attenuate the electric field [18]. In this way, it is easy to understand the affect the locations of the buried graphene on the loss performance of the hybrid waveguide.

## 3. Device Fabrication and Characterization

**Figure 3:**
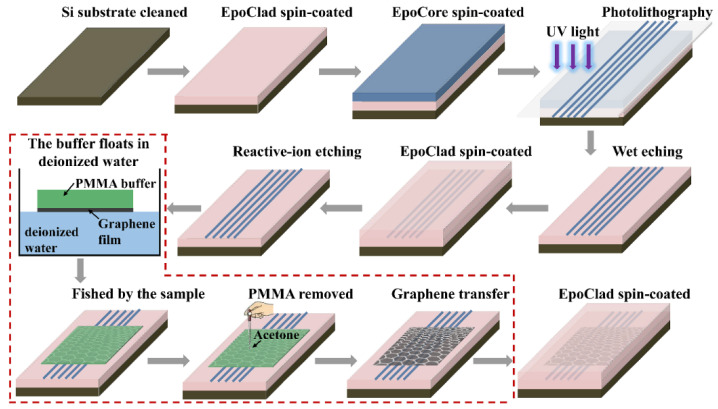
The fabrication process of the polarizer with graphene film placed on the surface of core layer.

**Figure 4:**
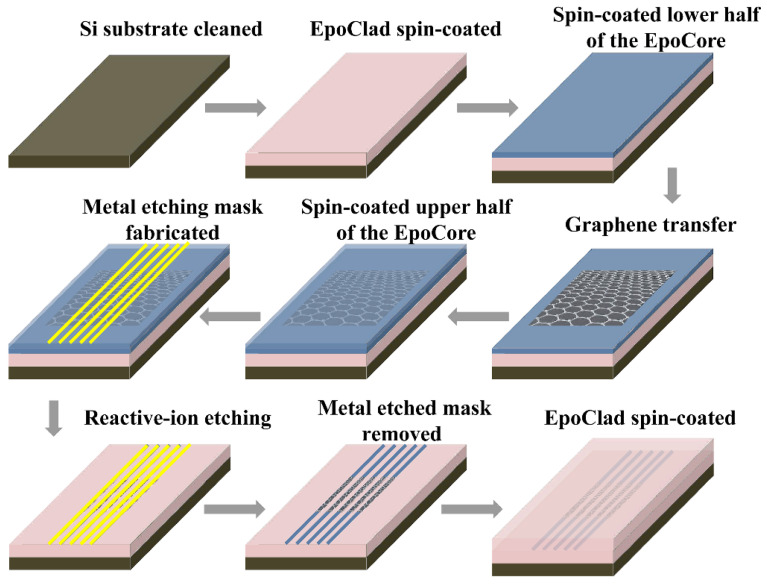
The fabrication process of the polarizer with graphene film buried in the center of core layer.

**Figure 5:**
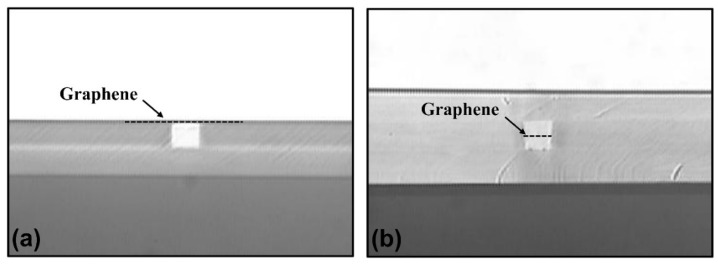
Cross sections of two fabricated polarizers, which incorporate, respectively, the graphene film was placed (**a**) on the surface of core layer and (**b**) buried in the center of core layer.

The designed polarizers were fabricated by using standard microfabrication techniques, wherein polymer materials EpoCore and EpoClad (Micro Resist Technology, GmbH, Berlin, Germany) were chosen as the core and cladding materials, respectively. Figure 3 shows the fabrication process of the polarizer with Structure 1 presented in Figure 1a. Firstly, an EpoClad film was spin-coated onto the Si substrate and fully cured to form a 10-µm thick under-cladding. For Structure 1, a 4-µm-thick EpoCore film was next spin-coated on the cured under-cladding to form the core layer, and then the waveguide cores with the desired widths were formed by the standard photolithography and wet-etching process. The waveguide cores were then covered by spin-coating EpoClad onto the sample. After EpoClad upper-cladding was fully cured, the upper-cladding layer on top of the core layer was etched away by reactive-ion etching (RIE) method to expose the core surface. Then, the wet-transfer technique process for graphene film was carried out, as shown inside the red dotted line in Figure 3. Firstly, the monolayer graphene film attached on the polymethylmethacrylate (PMMA) buffer with a dimension of 10 mm × 10 mm was floated into deionized water and fished by the sample to enable the graphene film to be transferred onto the waveguide core surface. Secondly, the PMMA buffer was dried and removed from the graphene film with acetone. Finally, a 8-µm-thick EpoClad film was spin-coated onto the sample to form the upper-cladding. For Structure 2 presented in Figure 1b, the fabrication scheme is shown in Figure 4. Firstly, the half of the core layer was spin-coated onto the prepared under-cladding. The monolayer graphene film with PMMA buffer was then transferred onto the sample according to the steps in Figure 3, and removed the PMMA buffer after being dried. Secondly, another half of the core layer was spin-coated onto the sample and fully cured. Finally, the rectangular cores buried with graphene layer was formed by photolithography and RIE process, and then covered with the EpoClad upper-cladding. Figure 5a shows the micrograph of the waveguide cross-section of the typical fabricated device for Structure 1 without upper cladding. Figure 5b micrograph of the waveguide cross-section of the fabricated device for Structure 2. Moreover, reference waveguides with the same dimensions and without integrating the graphene film were also fabricated on the same sample to examine the loss induced by the graphene film.

**Figure 6:**
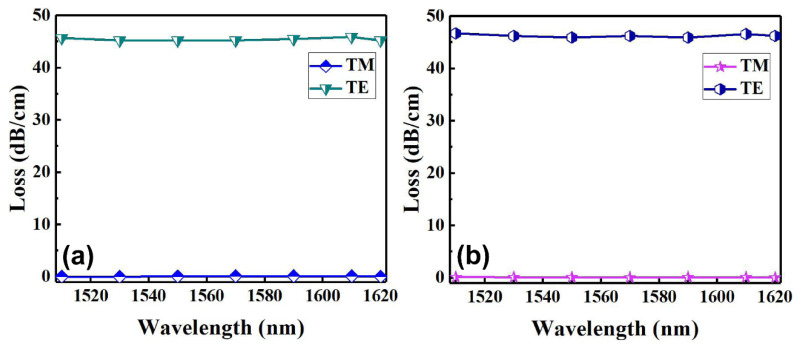
Graphene-induced losses measured for (**a**) Structure 1 and (**b**) Structure 2, respectively, in the wavelength range of 1510~1620 nm.

To characterize the performance of the fabricated polarizers based on the graphene-buried polymer waveguides, we used the polarization optics to control and monitor the polarization states of the input light and the output light. The input signal light from a semiconductor tunable laser (TSL-510, Santec, Aichi, Japan) is coupled directly into the input port of the polarizer through a lensed single-mode fiber (SMF) with a mode-field diameter of 2.5 µm. The output light beam from the polarizer can be collected with a lens, which images the output near-field patterns, and observed by an infrared charge-coupled device (CCD) camera. The images can be displayed on a video monitor during the testing process. After finding the output pattern, the output light was coupled into an optical power meter through a SMF to measure the output optical power from the polarizer and calculate the loss induced by graphene film and the insertion loss of the polarizer. By comparing the losses measured for the graphene-buried waveguide and the reference waveguide without graphene film, the losses induced only by the graphene films can be obtained. The variation of graphene-induced losses of the polarizers with the operating wavelength is indicated in Figure 6. Within a broad bandwidth over 110 nm in the telecommunication wavelengths, the polarizers present low loss and large extinction ratio (the difference between the graphene-induced losses for fundamental TM and TE modes), which are more than 45.1 dB and insensitive to the wavelength.

**Figure 7:**
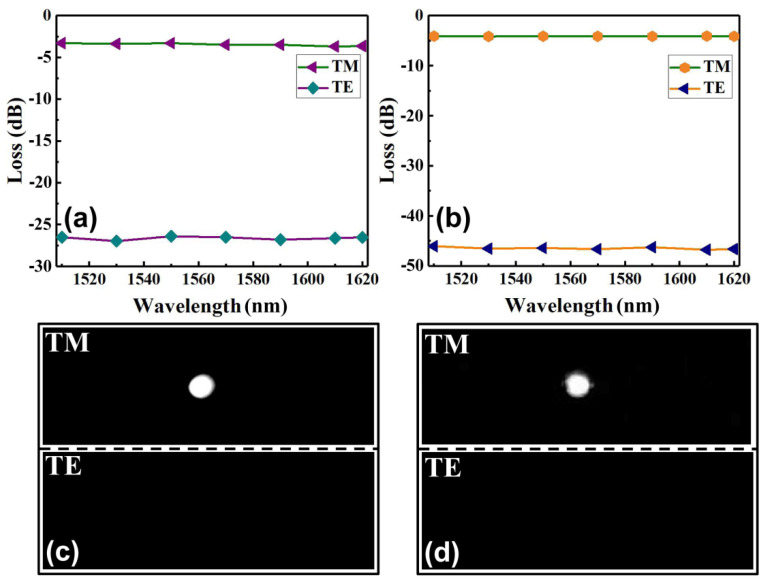
The insertion losses measured for (**a**) Structure 1 with a graphene film length of 3.6 mm and (**b**) Structure 2 with a graphene film length of 3 mm; the output near-field images taken at TM and TE polarization, respectively, for (**c**) Structure 1 and (**d**) Structure 2.

In fact, we should get a larger extinction ratio, however, this is limited by the measuring system rather than the graphene layer. Therefore, we dissociated the samples and further experimentally measured the loss performance of the polarizers embedded with a shorter length of graphene film. As shown in Figure 7a, through a single cleavage, we obtained Structure 1 with the embedded graphene film length of 3.6 mm and measured the insertion losses of the device under the TE and TM polarization, respectively. This polarizer presents an extinction ratio of more than 22.9 dB within a broad bandwidth over 110 nm and reached 23.6 dB at 1550 nm. That is, the monolayer graphene-induced loss has a good agreement with the simulating results for a graphene length shorter than 7~8 mm, at which the loss reaches our measurement limit of ~50 dB. Figure 7b shows the measured insertion losses of the polarizer for Structure 2 with the embedded graphene film length of 3 mm. The polarizer presents an extinction ratio of more than 41.9 dB within a broad bandwidth over 110 nm, and reached 42.3 dB at 1550 nm, which has a good agreement with the simulating results for the graphene length shorter than 3~4 mm. Figure 7c,d show the output near-field images of the fundamental modes for the proposed two structural polarizers obtained at 1550 nm under the TE and TM polarization, respectively. It can be seen that the TE mode was almost completely attenuated by the embedded graphene film, however, the TM mode was almost not influenced.

**Figure 8:**
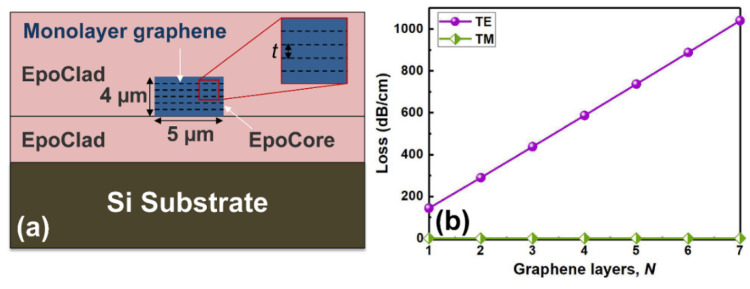
(**a**) The schematic diagram of the polarizer with different layers of graphene buried in the center of core layer and (**b**) the variation of the graphene-induced losses with the number of graphene layers.

Moreover, we further theoretically verified the effect of the number of the buried graphene layers on the fundamental mode losses. Figure 8a shows the schematic structure of polarizer with different layers of graphene buried in the center of waveguide core. Here, *n* is the number of graphene layers and *t* is the thickness of EpoCore between graphene sheets, where the *t* is 100 nm in our design. The variation of the graphene-induced loss of the polarizer with the numbers of graphene layers is shown in Figure 8b. We can see that with the graphene layers increase, the modal loss induced by the graphene gradually increases for the TE polarization, while the TM mode presents a lower loss, within a broad bandwidth. Therefore, we can increase the graphene layers to develop a polarizer with compact size and high extinction ratio, which is helpful to integrate with other polymer-based photonic devices. In addition, lower insertion loss can be achieved by selecting low-loss polymer materials.

## 4. Conclusions

In conclusion, to develop the polymer integrated waveguide polarizer in the optical communication system applications, we have proposed and experimentally demonstrated an ultra-broadband TM-pass polarizer based on polymer waveguide embedded with graphene layer. Polarizers with the graphene film buried in different locations are designed and fabricated to demonstrate the effects of the buried locations as well as the layers of the graphene on their loss performance. Our typical fabricated polarizer presents an ultra-high extinction ratio of more than 22.9 dB and 41.9 dB for the monolayer graphene placed on the surface of core layer and buried in the center of core layer, respectively, and as low insertion loss as ~4.0 dB for the TM mode with the bandwidth over 110 nm at telecommunication wavelengths. Furthermore, we can increase the graphene layers buried in the center of core to develop a polarizer with compact size and higher extinction ratio. The proposed polymer waveguide polarizers with high extinction ratio, low insertion loss, ultra-broadband, and easy fabrication process have potential applications for on-chip photonic integrated circuits.

## Data Availability

The data presented in this study are available upon request.
